# Production of Martian fiber by in-situ resource utilization strategy

**DOI:** 10.1016/j.isci.2024.110408

**Published:** 2024-06-28

**Authors:** Ze-Shi Guo, Dan Xing, Xiong-Yu Xi, Cun-Guang Liang, Bin Hao, Xiaojia Zeng, Hong Tang, Huaican Chen, Wen Yin, Peng Zhang, Kefa Zhou, Qingbin Zheng, Peng-Cheng Ma

**Affiliations:** 1 Laboratory of Environmental Science and Technology, The Xinjiang Technical Institute of Physics and Chemistry, Key Laboratory of Functional Materials and Devices for Special Environments, Chinese Academy of Sciences, Urumqi 830011, China; 2 Center of Materials Science and Optoelectronics Engineering, University of Chinese Academy of Sciences, Beijing 100049, China; 3 Center for Lunar and Planetary Sciences, Institute of Geochemistry, Chinese Academy of Sciences, Guiyang 550081, China; 4 Institute of High Energy Physics, Chinese Academy of Sciences, Dongguan 523000, China; 5 Spallation Neutron Source Science Center, Dongguan 523803, China; 6 Technology and Engineering Center for Space Utilization, Chinese Academy of Sciences, Beijing 100094, China; 7 School of Science and Engineering, The Chinese University of Hong Kong, Shenzhen, Shenzhen 518172, China

**Keywords:** Physics, Space sciences, Materials science

## Abstract

Many countries and commercial organizations have shown great interest in constructing a Martian base. *In situ* resource utilization (ISRU) provides a cost-effective way to achieve this ambitious goal. In this article, we proposed to use Martian soil simulant to produce a fiber to satisfy material requirement for the construction of Martian base. The composition, melting behavior, and fiber forming process of the soil simulant was studied, and continuous fiber with maximum strength of 1320 MPa and elastic modulus of 99 GPa was obtained on a spinning facility. The findings of this study demonstrate the feasibility of ISRU to prepare Martian fiber from the soil on the Mars, offering a new way to obtain key materials for the construction of a Martian base.

## Highlights

• *In situ* resource utilization is essential for constructing a Martian base
• Feasibility of using Martian soil to produce fiber material is demonstrated
• Martian fiber with a denser structure exhibits better mechanical performance
• Martian environment facilitates the preparation of high-performance fiber

## Graphical abstract

**Figure:**
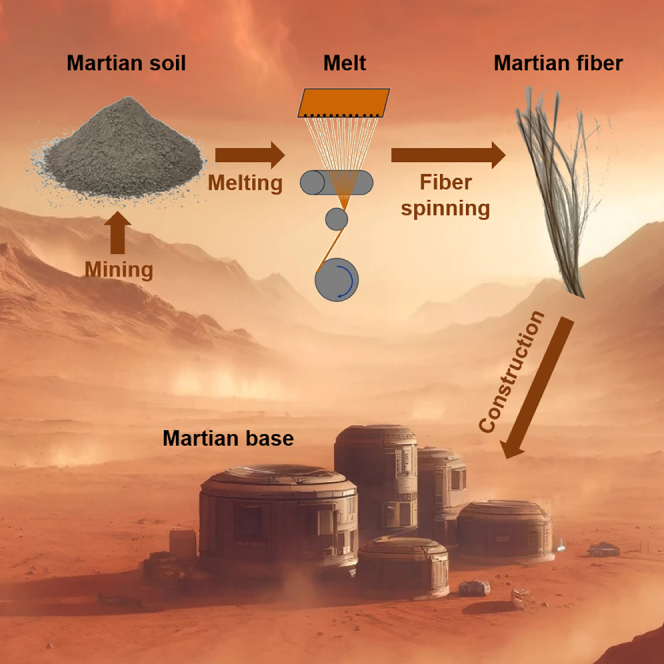

## Introduction

Mars is the fourth planet from the Sun and exhibits significant similarities to the Earth. It is considered to be the most suitable site for human migration in the solar system as well as a transit station for deep space exploration.^1^^,^^2^ Since the cold war, the United States and the former Soviet Union launched probes to Mars and put forward the idea of a Martian base with some prospective studies.^1^^,^^2^^,^^3^^,^^4^ More recently, NASA’s Perseverance rover landed on Martian surface and successfully flew a helicopter for survey.^5^ NASA plans to start the first human mission to Mars after finishing the Artemis program, which is expected to be the preparation for the human Mars exploration, aiming at constructing a lunar camp that can be used to launch and supply spacecraft to Mars.^6^ China launched its first Mars probe, Tianwen-1, with an orbiter, a lander, and a rover in 2020. The rover Zhurong has landed on Mars and started a serial of new exploration activities.^7^ China also set up a base to simulate the environment of Mars for future research.^8^ While there is no exact timetable for the construction of Martian base proposed by the leading countries, they all show great interest in achieving this giant ambition. Some commercial organizations have also made aggressive plans for the Martian base construction. For example, SpaceX proposed to build a city and send 1 million people to Mars by 2050. Blue Origin also expressed an interest in building a base on Mars.^9^ From the available information, it seems that the construction of a Martian base is only a matter of time.

The construction of a Martian base requires huge amounts of building materials. Considering the high cost and long distance from Earth to Mars, it is impossible to transport all these materials from the former. So how to develop a manufacturing system on Mars is one of the most challenging disciplines in both scientific and engineering fields.^10^ and *in situ* resource utilization (ISRU) is an effective way to address this. Mars’ surface is covered by a layer of soil, and the abundant amount of such soil makes it ideal for ISRU. As Martian soil is not available on Earth so far, the corresponding simulants, such as Johnson Space Center Mars Simulant (JSC Mars-1), Mars Global Simulant (MGS-1), and Jining Martian Soil Simulant (JMSS-1), are developed in recent years and used as a substitute for ISRU studies.^1^^,^^2^^,^^11^ These simulants are formulated by using Earth minerals based on the composition results of real Martian soil as detected and analyzed by various rovers and orbiters. For example, JSC Mars-1, one of the earliest Martian soil simulations developed by the NASA Johnson Space Center, is compounded by the glassy volcanic ash and cinder on Mauna Kea volcano in Hawaii.^12^ The simulant has a similar mineralogy and chemical composition to the Martian soil at the Viking and Pathfinder landing sites. This ensures that the simulated sample exhibits similar properties to the real Martian soil, such as spectroscopic characteristics, particle size distribution, and thermophysical properties, and can be replaceable for scientific research.^11^ By using various Martian soil simulants, different techniques were developed to explore the feasibility of ISRU on Mars.^1^^,^^2^^,^^13^^,^^14^^,^^15^^,^^16^ The simplest way is to sinter or melt the Martian soil. For instance, Warren et al. studied the effect of sintering temperature on the mechanical properties of Martian simulant and found that the optimal sintering temperature was in the range of 1100–1200^o^C; the obtained sample showed a compressive strength of 25 MPa.^14^ It should be mentioned here that the molding technique usually needs water as a binder for pre-molding, which is a rare resource on Mars. To solve this problem, researchers used Martian soil to make concrete or concrete-like products. A recent study reported the development of Martian concrete made from sulfur and magnetite (minerals enriched in Martian soil) by using microwave heating technique.^15^ As water is not required to process the concrete in this method, thus making it more suitable to satisfy the high temperature and low air pressure environment on Mars. The resistance of the developed material to the thermal cycling should be considered during the ISRU as cracks could be caused in the system due to the significant differences in the thermal expansion coefficient of the sulfur (minimal 35 × 10^−6^ K^−1^) and aggregate (8.4 × 10^−6^ K^−1^). Researchers also studied techniques to obtain metals from the Martian soil.^16^ Thermodynamic calculations indicated that reacting Martian soil simulant with carbon (at about 10 wt % addition) at 1120°C and 7 mbar would result in the formation of a liquid Fe alloy with a high conversion rate of 99.9%. Predictions showed that the primary impurities in the liquid Fe alloy would be Si, C, Cr, and P. This process was expected to generate a hot gas predominantly composed of carbon monoxide (CO), which could be employed to preheat the simulant. The CO could also be captured and condensed to recover carbon for reuse in the carbothermic reduction process.

While much progress has been achieved in this field with varying degrees of success, the current studies for the ISRU of Martian soil often focused on materials with bulky structures, like concrete or metal. With consideration on the harsh environment on Mars, these traditional materials may not be versatile enough to satisfy the structural and functional requirements of material for base construction. In this context, developing novel materials with tailored performance is highly desirable. One of the ideal materials is composites, like the steel-reinforced concrete, a common construction material on Earth. Composites are a binary system consisting of a matrix and reinforcement.^17^ Usually, the reinforcement has better mechanical properties and plays a key role in governing the performance of composites, whereas the matrix in the composites is responsible for bonding the reinforcement and protecting it from the environment.

Basalt fiber is a kind of inorganic filament made from basalt rock. This fiber has attracted great attention as reinforcement in construction material on Earth.^18^ Martian soil has a similar composition and mineralogy to the basalt on Earth,^19^^,^^20^^,^^21^ so if we could use such soil to produce corresponding fiber, and the obtained material had the potential to be used as reinforcement for the construction on Mars. In other words, combination of Martian fiber with corresponding soil provides a new way to address the material challenges for the construction of Mars base.

Some groups and organizations have put forward the concept of a Mars base, however, marginal progress has been made so far focusing on offering material solution for this. The purpose of this article is to demonstrate the feasibility of producing Martian fiber (MF) using corresponding soil, and evaluate the mechanical performance of the obtained fibers for providing key materials for the construction of base on Mars.

## Results

**Figure 1:**
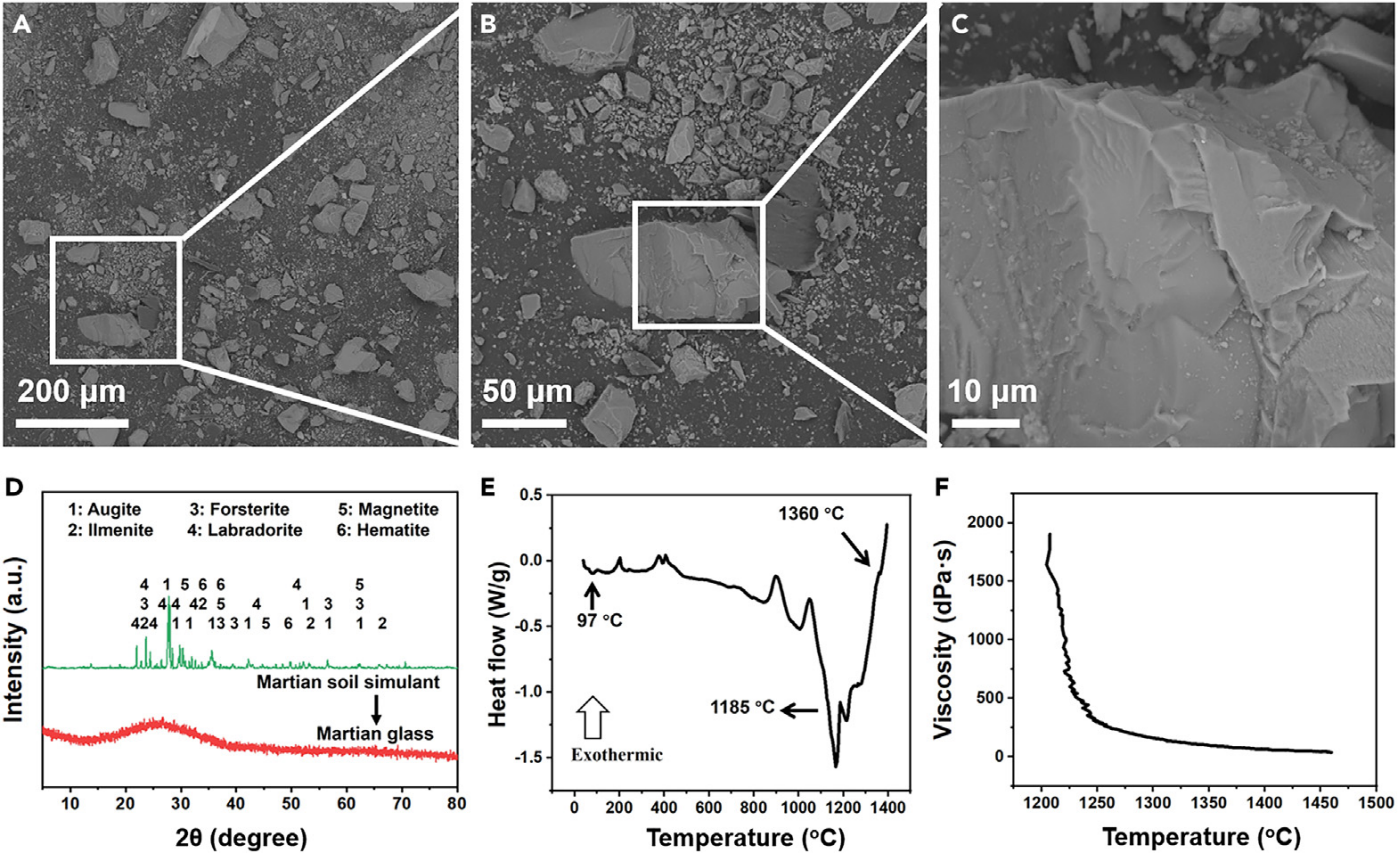
Morphology and properties of Martian soil simulant (A–C) SEM images under different magnifications. (D) Mineral phases of simulant and XRD spectra. (E) DSC curve of the sample. (F) Temperature-viscosity curve of the sample.

The initial study was focusing on getting information on the properties of Martian soil simulant. Images from the scanning electronic microscope (SEM) confirmed that the grain size of this simulant is below 1 mm (Figure 1A), and the mean particle size is about 250 μm. The typical particle in the sample showed a rough and uneven granular surface with many edges and corners (B and C in Figure 1), which is the result of a mechanical comminution process. This process was close to the physical weathering on Mars, where wind abrasion and meteoric impact led to the formation of the Martian soil.^21^ The simulant has a similar composition to the Martian basaltic soil (Table 1).^21^ In both samples, SiO_2_ is the major composition with around 50% of weight, followed by Al_2_O_3_, Fe_x_O_y_, MgO and CaO. In fiber science and technology, the acidity modulus (*M*_*k*_), as defined by the *(W*_*SiO2*_*+W*_*Al2O3*_*)/(W*_*MgO*_*+W*_*CaO*_*)*, is used to evaluate the possibility of material to form filament, and the optimal *M*_*k*_ for continuous basalt fiber is in the range of 3.0–6.0.^22^ The calculated *M*_*k*_ for the average Martian soil and simulant are 3.7 and 4.1, respectively, suggesting the possibility of the idea on turning Martian soil into fiber.

**Table 1:** Comparison on the chemical composition of Martian soil and the simulant

Composition (wt %)	SiO_2_	Al_2_O_3_	Fe_x_O_y_	MgO	CaO	Na_2_O	K_2_O	TiO_2_	MnO	P_2_O_5_
Soil average	45.41	9.71	16.73	8.35	6.37	2.73	0.44	0.91	0.33	0.83
Simulant	49.83	13.83	15.41	6.79	8.68	2.76	0.99	1.70	0.01	0.01

The crystal phase in the simulant is illustrated by an X-ray diffractometer (XRD), and it is found that the material is a mixture of several minerals (Figure 1D), such as augite (Ca(Mg,Fe,Al)[(Si,Al)_2_O_6_], PDF#01-073-8541), ilmenite (FeTiO_3_, PDF#04-006-6575), forsterite (Mg_2_SiO_4_, PDF#97-006-4739), labradorite ((Na,Ca)(Al,Si)_4_O_8_, PDF#04-011-6816), magnetite (Fe_3_O_4_, PDF#04-006-6550) and hematite (Fe_2_O_3_, PDF#04-003-2900). These results were in good agreement with those obtained from launched probes in real Martian soil.^21^ The XRD result of the quenched sample shows a broad hump in the 2θ range of 20°–30° (Figure 1D), indicating the transformation of crystal structures in the sample to the amorphous state.

Differential scanning calorimetry (DSC) technique gives information on the thermal behavior of soil under elevated temperature. The first endothermic peak at approximately 100°C is attributed to the volatilization of water adsorbed in the sample, and the last one at around 1360°C means the complete melting of the sample under the elevated temperature (Figure 1E). Interestingly, several exothermic peaks are also noticed in the sample, suggesting the crystallization or oxidation of crystals in the melt. For instance, the one at 1185°C is attributed to the formation of the hematite in the melt.^23^ Above this temperature, there will be absence of crystals in the material, suggesting the formation of entirely glassy state of material. The viscosity of the simulant was significantly decreased from 1500 to 250 dPa s within a narrow temperature range of 1210°C–1260°C (Figure 1F). It kept nearly constant above 1350°C, meaning the complete melting and homogenization of the sample. This result along with DSC data gave a reference on controlling the temperature for the fiber spinning. For the real Martian soil, besides the basalt, there are many sedimentary rocks, such as phyllosilicate, sulfate and evaporate.^1^^,^^2^ The presence of these materials will definitely affect the melting behavior of the soil and produce gases at high temperature. Under such circumstances, proper control on the temperature is important to ensure the homogeneity of the melted soil. Because incomplete melting of soil or gas residue will result in the presence of defect in the fiber, which definitely decreases the mechanical performance of the fibers.

**Figure 2:**
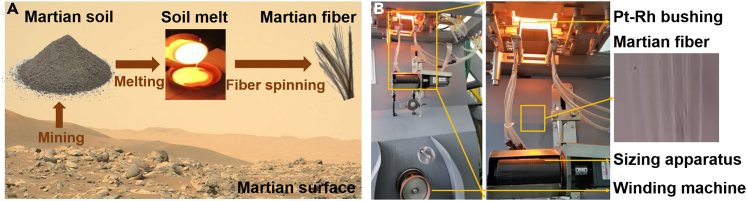
Experimental setups for the production of Martian fiber (A) Technical design of a two-step procedure for fiber spinning, and the pictures are the states of material at different experimental setups. (B) Facility for the production of continuous fiber.

**Figure 3:**
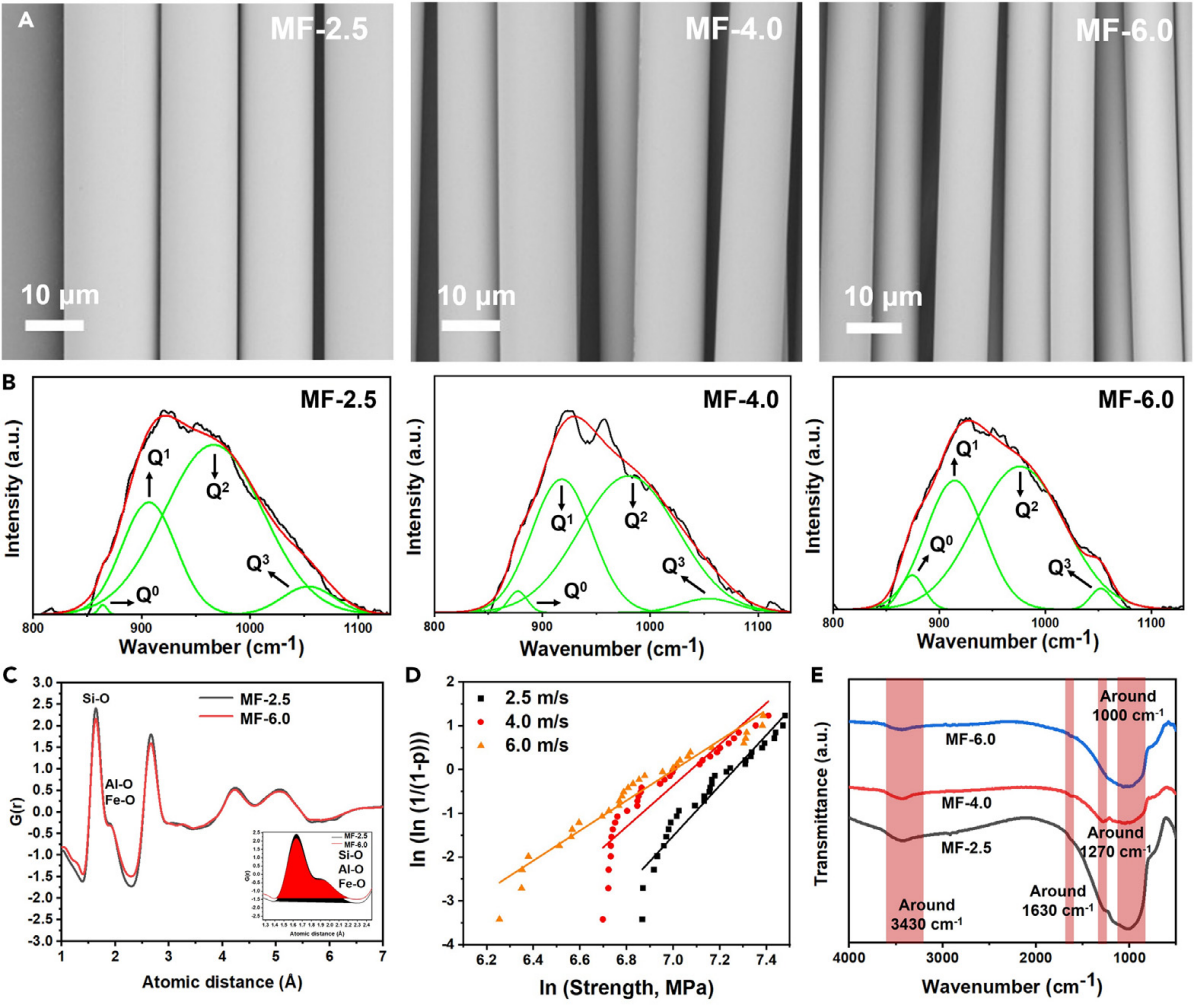
Properties and fine structures of MF produced at the different winding speeds (A) Morphology. (B) Raman spectra and fitting results, for all fitting results *R*^*2*^ > 0.99. (C) Partial distribution function. (D) Weibull distribution of tensile strength. (E) FT-IR spectra.

A two-step procedure was established to produce the continuous MF (Figure 2 and Experimental procedures). The morphology of MF was visualized by SEM. From the SEM images (Figure 3A), we can see that the surface of all fibers is smooth and cylindrical. This observation originated from the melt shrinkage under the effect of surface tension during the fiber spinning. The diameter of the fiber decrease from 13.9 to 9.7 μm with increasing winding speed (Table 2), this is due to the fact that the melt undergoes a rapid lengthening and thinning process, and this is accompanied by the solidification and external traction during the spinning.

**Table 2:** Tensile properties of MF produced at the different winding speeds

Sample	Winding speed (m/s)	Fiber diameter (μm)	Tensile strength (MPa)	Tensile modulus (GPa)	Elongation at break (%)	Weibull modulus
MF-2.5	2.5	13.9 ± 1.4	1320 ± 249	99 ± 23	1.5 ± 0.4	5.77
MF-4.0	4.0	11.5 ± 0.6	1081 ± 247	94 ± 20	1.3 ± 0.3	4.71
MF-6.0	6.0	9.7 ± 0.8	990 ± 317	94 ± 15	1.1 ± 0.3	3.44

The tensile strength of the fiber decreased from 1320 to 894 MPa with increasing winding speed, whereas the moduli of fibers were maintained at around 94 GPa. The main difference between the Martian fiber made in this study and the Earth counterpart is the chemical composition. Specifically, Martian fiber has a higher iron content (16.73 wt % of Fe_x_O_y_) than that of commercial Earth basalt fiber (usually around 10 wt % of Fe_x_O_y_). We found that the tensile modulus of MF (from 94 to 99 GPa) is higher than most commercial basalt fibers (Typically around 80 GPa). This may be because of the higher iron content in JMSS-1. As Fe^3+^ can be a network former in the fiber,^23^ thus forming a tightly packed glass with a denser structure, and this leads to a higher elastic modulus of the filament. It should be mentioned here that when following the size-effect theory caused by the surface defects in a brittle material,^24^ the strength of the fiber should be increased with decreasing diameter due to the decrease of the defect size and occurrence probability. We speculated that such deviation was caused by the microstructure of fiber under different winding speeds. The fiber is in an amorphous state and the atoms in the material are arranged in a long-range disordered way and stacked as dense as possible to form a random stacking microstructure.^25^ Therefore, the denser microstructure results in an enhanced tensile strength of fiber. In addition, during the fiber spinning process, the lower winding speed resulted in a larger diameter of the fiber, and the cooling rate of the outside fiber was much higher than that in the inside. In this way, the atoms in the fiber have a longer time for relaxation, which leads to a denser microstructure.^26^

## Discussion

To prove the above assumption, Raman spectroscopy was used to evaluate the fine structure of fiber samples. The broad band in the range of 800–1200 cm^−1^ reflects the symmetric stretching vibration of Si-O tetrahedron, and this position was mainly composed of four spectral peaks caused by the symmetrical stretching vibration of bridging oxygen in terms of *Q*^*0*^, *Q*^*1*^, *Q*^*2*^, and *Q*^*3*^, corresponding to the Raman shifts at around 850-880 cm^−1^, 900-920 cm^−1^, 950-980 cm^−1^ and 1050-1100 cm^−1^, respectively.^27^ The *Q*^*n*^ is classified by the number of bridging oxygen in the Si-O tetrahedron, for example, *Q*^*3*^ stands for silicon coordinated by three bridging oxygen atoms, and *Q*^*0*^ means the absence of bridging oxygen. The relative percentages of various structural units were analyzed by Gaussian fittings as shown in Figure 3B, and calculated using the fitted peak areas as expressed in Equation 1:^28^

(Equation 1) $$Xn=\frac{An/Sn}{\sum_{0}^{3}An/Sn}$$

Where *X*_*n*_ is the relative percentage of various structural unit *Q*^*n*^, *A*_*n*_ is the percentage of fitted peak area of *Q*^*n*^, and *S*_*n*_ is the Raman scattering coefficient of *Q*^*n*^ (Here *S*_*0*_ = 1, *S*_*1*_ = 0.514, *S*_*2*_ = 0.242, *S*_*3*_ = 0.09).^28^

The ratio of relative percentage of *Q*^*3*^ to *Q*^*2*^ (*X*_*3*_*/X*_*2*_) was used to describe the degree of polymerization of glass, and the larger the ratio is, the denser the glass structure presents.^29^ By comparing the *X*_*3*_*/X*_*2*_ ratio of different samples, we can evaluate the packing compactness of microstructure in fiber sample. As shown in Table 3, the MF produced at 2.5 m/s has the highest *X*_*3*_*/X*_*2*_ ratio, and the ratio decreased with increasing winding speed, confirming that the fiber produced at a lower winding speed has a denser microstructure, this in turn leads to a higher tensile strength of the fiber.

**Table 3:** Relative content of *Q*^*n*^ of MF produced at the different winding speeds

Sample	*X*_*0*_	*X*_*1*_	*X*_*2*_	*X*_*3*_	*X*_*3*_*/X*_*2*_	Average strength (MPa)
MF-2.5	0.09	13.61	60.32	25.98	0.43	1320
MF-4.0	1.54	23.56	66.48	8.42	0.13	1081
MF-6.0	1.77	22.79	70.85	6.61	0.09	990

The pair distribution function (PDF) data were collected from the neutron scattering source to further investigate the effect of winding speed on the microstructure of fiber. As shown in Figure 3C, the first peak appeared in PDF for the two typical fibers (MF-2.5 and MF-6.0) is around 1.64 Å, which reflects the intra-tetrahedral Si-O distance. The peaks between 1.7 and 2.0 Å correspond to the distances of other glass network formers with oxygen, such as Al-O and Fe-O.^30^^,^^31^ As we can see from the curves, the value of PDF (*G*_*r*_) of glass network former in MF-2.5 sample is always higher than that of the MF-6.0, meaning a tighter network formed by the tetrahedral structure in the former sample. Additionally, we calculated the peak area (Inset in Figure 3C) of the PDF, and found that this value for MF-2.5 (1.31) is 15% higher than MF-6.0 (1.11). Since the fibers produced at different winding speeds have the same chemical composition, we can determine the degree of network aggregation by directly comparing the peak areas, and a higher peak area represents a higher average *Q*^*n*^,^32^^,^^33^ which is consistent with Raman results.

The Weibull plots of tensile strength for single MF samples produced at different winding speeds are shown in Figure 3D, and the Weibull modulus are summarized in Table 2. The Weibull modulus decreased with an increasing winding speed. This parameter can be used to characterize the probability of defect occurrence on the fiber surface. A higher Weibull modulus indicates that the fiber has a lower probability of failure, which is attributed to the denser structure of the fiber.^34^ In other words, the denser structure of MF has a higher resistance to the physical damage (such as scratch) during the fiber spinning, making the defects on the fiber surface distribute more uniformly, thus leading to a higher tensile strength for the filament.

From the FT-IR curves of MF (Figure 3E), we can see that all fiber samples show a pretty similar pattern. Specifically, the wide band centered at 3430 cm^−1^ and the one at around 1630 cm^−1^ correspond to the stretching vibration of hydroxyl group,^35^ and the strong band at around 1000 cm^−1^ is ascribed to the anti-symmetric vibration of Si-*O*-Si in the material.^36^ The one at around 1270 cm^−1^ indicates the stretching vibration of Al-*O*-Si network.^35^ It should be mentioned here that the hydroxyl groups on the fiber surface can adsorb and react with polymeric, ceramic or metallic matrices. Although no polymeric or metallic matrices exist on Mars, researchers have shown that it is possible to convert atmospheric CO_2_ into polymers,^37^ and metallic materials can be extracted from the minerals.^13^ Therefore, the Martian fiber developed in this study has great potential as reinforcement to develop composites for the construction of a Martian base.

The environment of Mars is different from that of the Earth’s. Mars has a low gravity (1/3 g) and atmospheric pressure (0.7 kPa), high radiation environment, and unique atmosphere (95.0% CO_2_, 3.0% N_2_, 1.6% Ar and 0.13% O_2_).^1^ Such conditions will affect the fiber formation. For the low gravity, previous studies reported that the structure of glass/glass fiber prepared under low gravity (0.01g) was more homogeneous, and exhibited higher resistance for the crystallization than the one prepared on Earth.^38^^,^^39^ The enhanced homogeneity of constituents can improve the uniformity of fiber and results in better mechanical performance. Whereas the improved resistance to the crystallization brings a positive effect on improving the continuity of fiber and a more straightforward design for equipment. Due to the weak Mars atmosphere, the average radiation level on Mars surface reached 100 mSv, much higher than the average radiation level on Earth (2.4 mSv).^1^ Such high radiation environment presents challenges in designing, manufacturing, and maintaining the equipment for fiber production. At the same time, a high radiation environment may affect the performance of materials. In the case of basalt fiber (similar to MF), it has been experimentally demonstrated that there was a loss of mass due to the volatilization of molecules adsorbed on the fiber surface under gamma-irradiation (150 kGy), resulting in a slight increase in the filament strength. However, marginal variation on the composition of the fiber was observed upon the radiation.^40^ Anyway, the MF was expected to exhibit better radiation resistance on Mars when comparing with that of the polymeric materials.^1^^,^^41^

The Mars atmosphere is mainly composed of CO_2_ (about 95%), and ferric iron (Fe^3+^) has a much higher abundance than divalent one (Fe^2+^) because the planet is red. The presence of Fe^3+^ in real Martian soil will facilitate the formation of fiber, as previous studies showed that the higher amount of Fe^3+^ in the basalt will decrease the material melting point.^23^^,^^42^ In addition, the fiber is absence to the moisture erosion in the dry Martian atmosphere, which brings a positive effect on maintaining the mechanical performance of the fiber. Besides the natural environment of Mars, there is a concern on the energy consumption and feasibility for the large-scale production of MF. The facility used in this study allowed the production of fibers by employing the bushing with 50 nozzles, and we believe that the productivity can be significantly enhanced by using bushing with more nozzles. Our recent study showed that by heating the simulant using the microwave irradiation, the input energy for melting the same amount of Martian soil would be reduced by more than 85%.^43^ Anyway, the energy requirement for the larger-scale fiber production depends on several operational factors, such as the amount of soil to be melted, melting technology, as well as thermal management on the fiber spinning facilities.

### Conclusion

In summary, we demonstrated the feasibility of using Martian soil to get corresponding fiber. Based on the analysis on the chemical composition and properties of soil simulant, continuous fiber was produced under different winding speeds on a 50-hole fiber spinning facility. The fiber produced with a winding speed of 2.5 m/s shows the highest tensile strength of 1320 MPa, closing to the fiber prepared on Earth used for the development of composite materials. The findings of the current study confirm that continuous fibers can be obtained from Martian soil by melting and spinning, and the results offer a new way for the *in situ* utilization of Martian resource to develop structural and functional materials for the construction of Martian base.

There will be numerous challenges in achieving this goal, such as the influence of the Martian environment (Low gravity, inert atmosphere, etc.) on the fiber properties, design and system integration for fiber spinning equipment. Since it is difficult to simulate the Martian environment for the experiment on Earth, further demonstration tests can be carried out at the space station to investigate the properties of fibers obtained under different environments. The design and optimization on the fiber-forming equipment, study on the interfacial interaction between MF and matrix, will also be the challenging topics to realize the construction of Martian base. While future research may face a range of challenges in scientific and engineering aspects, there will be a bright future in this interesting field with the involvement of more researchers and collaborations.

### Limitations of the study

One of the primary limitations of this study was the fiber spinning process implemented in the Earth’s environment. The unique Martian environment could change the fiber spinning process and properties. However, this study does not conduct experiments to study the effects of the Martian environment on the fiber spinning process and properties due to the limitations of the experimental conditions. Only a theoretical analysis was made through references to discuss this problem. Furthermore, instead of directly observing the defects on the fiber surface through experiments, we compared the defect states on the fiber surface using the Weibull analysis. Nevertheless, the Weibull analysis is widely used in strength analysis of brittle materials, and its Weibull modulus can be used to characterize the defect of the material.

## STAR★Methods

### Key resources table

**Table:** 

REAGENT or RESOURCE	SOURCE	IDENTIFIER
**Deposited data**		

JMSS-1 Martian regolith simulant	Institute of Geochemistry, Chinese Academy of Sciences	https://doi.org/10.1186/s40623-015-0248-5
Properties of JMSS-1	This paper	Section ‘‘results’’
Properties of Martian fiber	This paper	Section ‘‘results’’

**Software and algorithms**		

JADE	MDI	https://www.materialsdata.com/prodjd.html
Origin	OriginLab	https://www.originlab.com/index.aspx?go=PRODUCTS/Origin
General Structure Analysis System (GSAS) Ⅱ program	Toby et al.^44^	https://doi.org/10.1107/S0021889813003531

**Other**		

Scanning electron microscopy (SEM)	Phenom Scientific	Phenom XL
X-ray fluorescence (XRF)	Bruker	S8 TIGER
X-ray diffraction (XRD)	Bruker	D8
Differential scanning calorimetry (DSC)	Netzch	STA449F3
High-temperature rotary viscometer	Brookfield	DV-Ⅲ
Fiber tensile tester	Shanghai Xinxian	XQ-1A
Raman spectroscopy	Horiba	LabRAM HR Evolution
Fourier transform infrared spectroscopy (FT-IR)	Thermo Fisher	Nicolet iS50
Multi-physics Instrument established in China Spallation Neutron Source	Xu et al.^45^	https://doi.org/10.1016/j.nima.2021.165642

### Resource availability

#### Lead contact

Further information and requests for resources and reagents should be directed to and will be fulfilled by the lead contact, Peng-Cheng Ma (mapc@ms.xjb.ac.cn).

#### Materials availability

The source of materials is displayed in the key resources table.

#### Data and code availability

• Data: Data reported in this paper will be shared by the lead contact upon request.
• Code: This paper does not report original code.
• Any additional information required to reanalyze the data reported in this paper is available from the lead contact upon request.

### Experimental model and study participant details

The experimental model and study participant details are described in the main text (See method details section).

### Method details

#### Martian fiber production

In a typical process, Martian simulant (250.0 g) was put into a corundum crucible and melted in an electrical furnace to homogenize the starting material. The temperature was raised from room temperature to 1200°C at a heating rate of 5°C/min and then increased to 1500°C at a heating rate of 2°C/min. The melt was quenched by water to get the material in a glass state. Then the glass was crushed and added into the furnace of a fiber spinning facility established in our lab (Operation voltage 380 V, rated power 10 kw, Figure 2B). During the experiment, the melted glass at around 1250°C was continuously drawn from the platinum-rhodium alloy bushing with 50 nozzles, and water was used as a sizing agent to facilitate the winding of the filament. Continuous fibers with different diameters were obtained by controlling the speed of the winding machine with 2.5, 4.0 and 6.0 m/s, and the obtained fiber was dried in an oven at 120°C for 12 h.

#### Characterization

The morphology of materials was characterized by a scanning electron microscopy (SEM, Phenom XL, Phenom Scientific, US). The sample for SEM characterization was gold-plated to enhance the conductivity. The major element composition of material was analyzed by X-ray fluorescence (XRF, S8 TIGER, Bruker, Germany). The crystal and mineral phases in the material were characterized by X-ray diffraction (XRD, D8, Bruker, Germany). The change of mass and heat flow of Martian simulant was characterized by differential scanning calorimetry (DSC, STA449F3, Netzch, Germany). The viscosity of melt was obtained on a high-temperature rotary viscometer (DV-Ⅲ, Brookfield, USA). The tensile strength of single fiber was tested on a fiber tensile tester (XQ-1A, Shanghai Xinxian, China). The tests were performed according to the ASTM C1557-14 standard, the gauge length and loading speed were fixed at 25 mm and 2 mm/min, respectively. The microstructure of fiber was characterized by Raman spectroscopy (LabRAM HR Evolution, Horiba, Japan) with a green laser (532 nm). The functional groups on fiber surface were characterized by the Fourier transform infrared spectroscopy (FT-IR, Nicolet iS50, Thermo Fisher, USA). Neutron diffraction experiments were performed on a Multi-physics Instrument established in China Spallation Neutron Source, which is a total scattering neutron time-of-flight diffractometer.^45^ Structure refinement was performed using the General Structure Analysis System (GSAS) Ⅱ program based on the Rietveld method.^44^

### Quantification and statistical analysis

A two-parameter Weibull model was utilized to assess the distribution of single fiber strength, where the failure probability (*P*) of a fiber of length (*L*) breaking under an applied strength (*σ*) is described by Equation 2:

(Equation 2) $$\begin{array}{c} P=1-\exp\left[-\frac{L}{L_{0}}{\left(\frac{\sigma }{\sigma_{0}}\right)}^{m}\right] \end{array}$$

Where *m* represents the Weibull modulus (shape parameter), and *σ*_*0*_ denotes the characteristic fiber strength (scale parameter) at a reference length *L*_*0*_, defined as the longest fiber length that contains only one surface flaw. Typically, *L*_*0*_ is set to unity for simplicity. By applying the natural logarithm to Equations 2 and 3 is derived:

(Equation 3) $$\begin{array}{c} \ln\left(\ln\left(\frac{1}{1-P}\right)\right)=\text{mln}\left(\sigma \right)-\text{mln}\left(\sigma_{0}\right)+\ln\;\left(L\right) \end{array}$$

Plotting *ln(σ)* as a function of $\ln\left(\ln\left(\frac{1}{1-P}\right)\right)$, a straight line is expected, and the slope, *m*, is the Weibull modulus, and the characteristic fiber strength, *σ*_*0*_, can be obtained from the intercept of the line. Usually, the probability of fiber failures *P* is estimated by Equation 4:

(Equation 4) $$\begin{array}{c} P=\frac{i}{n\;+\;1} \end{array}$$

Where *n* is the number of fibers tested, and *i* is the rank of the data point, assigned based on the tensile strength of the sample in ascending order.
